# On the Doctrines Respecting the Structure and Functions of the Heart and Arteries, First Learned from Eustachio Rudio by William Harvey, and Which Were His Direct Guide to the Study, Recognition, and Demonstration of the Circulation of the Blood

**Published:** 1840-07

**Authors:** 


### ART. XVII.

*Delle Dottrine sulla Struttura e sulle Funzioni del Cuore e delle Arterie, che imparò per la prima volta in Padova Guglielmo Harvey da Eustachio Rudio, e come esse lo guidarano direttamente a studiare, conoscere, e dimostrare la Circolazione del Sangue. Disquisizione di* GIOVANNI MARIA ZECCHINELLI.—*Padova*, 1838. 8vo, pp. 100.

*On the Doctrines respecting the Structure and Functions of the Heart and Arteries, first learned from Eustachio Rudio by William Harvey, and which were his direct guide to the study, recognition, and demonstration of the Circulation of the Blood.* A Dissertation by GIOVANNI MARIA ZECCHINELLI.—*Padua*, 1838.

THIS is an elaborate and formal dissertation, in which an attempt is made to deprive Harvey of the greater part of his glory as the discoverer of the circulation, and to confer it on one of the author's countrymen, Rudio or Rudius. Our limits would not permit us to enter, at present, into the full merits of this discussion if so inclined; but in truth, amid all his special pleadings, the author himself affords sufficient authority for denying the conclusions which he pretends to arrive at. Rudius (Eustachio Rudio), it appears, succeeded Massaria in the chair of medicine in Padua in the year 1599, the year after Harvey's arrival, and continued teaching there during the whole period of his stay. (Harvey graduated in April, 1602.) Before his residence in Padua, and so far back as 1587, Rudius published his work, "De Virtutibus et Vitiis Cordis," and in 1600, during Harvey's stay at Padua, he printed, at Venice, that entitled "De Naturali atque Morbosa Cordis Constitutione." In this last he informs the reader that he had taught in his lectures the doctrines exposed in it, two months before its publication. Doctor Zecchinelli maintains that Harvey *must* have been acquainted with all the doctrines contained in these works of Rudius, both as auditor of his lectures and reader of the works themselves, which, from particular circumstances mentioned by him, excited much notice in the university at the time.

It is true that Harvey does not mention Rudius in his writings; but is not the very statement of our author, that it was Rudius who directed Harvey to the writings of Servetus, Columbus, and Cesalpinus, "*wherein he found much of what he had learned of Rudius more clearly stated*

*than by him*," a sufficient reason for this silence? It may indeed be true that Harvey was led by Rudius to consult these authors; but by Dr. Zecchinelli's own showing this is nearly all for which he could have been indebted to him; since he himself (Dr. Z.) admits that nearly all the truths that Harvey might have learned from Rudius, Rudius had himself learned from other authors; while these other authors contained many other truths of which his hero Rudius was ignorant. In fact this *Disquisizione* is a much greater exposure of the poverty of the Paduan professor than of our great countryman, as the subjoined abstract of his summary will show. Of this summary we will only further remark, that while it repeats all the charges previously made against Harvey, of borrowing or stealing from others, without adding a single new one, it displays such a self-evident and resolute determination to strip him of his honours, at all hazards, as to do away with the necessity of all defence. This, therefore, we shall not attempt; nor shall we stop to put the author right even where he is palpably wrong; but we confess we should not be sorry if this attack should stir up some of our young and zealous brethren to vindicate, in detail, the reputation of our greatest physiologist from every aspersion cast on it by this medical Zoilus.

"I. *Things false taught by Rudius to Harvey.* 1. Formation of blood in the liver: maintained by Harvey. 2. Passage of the blood through the ventricular septum: corrected by Harvey, but previously known to Berengarius, Vesalius, Servetus, and Columbus. 3. That the pulmonary artery conveys air to the left ventricle: corrected by Harvey, but previously known to Columbus. 4. That the spirits and carbon (fuligo) are generated in the left ventricle—the former escaping by the aorta, the latter returning through the pulmonary artery: derided by Harvey, but previously by Cæsalpinus. 5. That the spirits generated in the left ventricle flow over the whole body: refuted by Harvey, who, however, does not notice that Rudius himself had said that "spirituous blood" also flows.

"II. *Things true taught by Rudius.* 1. That the *vena arteriosa* (pulm. art.) has the office of an artery, and the arteria venosa (pulm. vein) that of a vein: claimed by Harvey, but previously noticed by Cæsalpinus. 2. The use of the valve of the heart in opening and closing to admit the passage and prevent the return of the blood and spirits or *spirituous blood*: Harvey *first* learned *this* from Rudius, and at the same time obtaining a knowledge of the existence of similar valves in the veins generally, inferred the functions of the latter. 3. The passage of the blood from the right ventricle to the lungs, not merely to *nourish* them, but for an *ulterior purpose*: this statement of *ulterior purpose* was concealed (dissimulato) by Harvey. 4. The transit of the *spirituous blood* through the arteries and whole body, to convey heat, life, nutrition: a statement passed over by Harvey, while he enlarged on the ancient error of the arteries containing *only spirit*. 5. Pulsation communicated by the coats of the arteries, not by their contents: reversed by Harvey.* 6. The having referred, though only slightly (ma leggermente), to vivisections, the ligature and section of vessels: followed out by Harvey, who was however incited thereto and supported by the previous statements of Columbus and Cæsalpinus, and by the *opportunities of his position!* 7. The having made a reference, however slight (un lievissimo cenno), of the communication between the arteries and veins in the liver: Harvey concealed that any other had spoken of this communication.

"III. *The Defects of Rudius were*: 1. He omitted to notice that the vena arteriosa (pulm. art.) is larger than is required for the mere nutrition of the

* This should have come into the former category of *Things false*.—REV.

lungs: Harvey speaks of this, but had learned it of Columbus if not of Servetus. 2. He did not state that the blood in the lungs passes from the arteries into the veins by direct communication: Harvey takes credit for this discovery, which, however, belongs to Servetus, and was still better exposed by Cæsalpinus. 3. He never spoke clearly of the blood in the arteries, confounding this with the spirits, heat, &c.: Harvey did, but the fact was known before him from the dissection of living animals.

"IV. *The essential things taught by Rudius and neglected by Harvey were:* 1, The influence of the heart on the affections of the mind; 2, the action of the nerves; 3, the peculiar nature of the fibres of the heart, &c."

Such is the case made out for Rudius,—a case which, as we have said, tells little in his favour, however it may tell against Harvey.

We subjoin, by way of curiosity, our author's enumeration of what he terms "*The real Merits of Harvey;*" which if they are generally and severally poisons to the vanity of his countrymen, he has assuredly done his best to provide antidotes against their evil working. The *catalogue raisonnée* is really amusing.

"1. Harvey recognized the use of the veins, but he did so by inference, after having been taught by Rudius the use of those of the heart:—a merit of *induction, not of Discovery!* 2. He practised vivisection, thereby ascertaining what *he* terms *new* and *unheard-of* things, but both the vivisections and these new and unheard-of things had been indicated (additate) by others:—a merit of *imitation, confirmation,* at most *extension,—not of Discovery!* 3. He proved that the whole mass of the blood passes through the heart, and in so short a space as could not be furnished by nutrition; and throughout the body continually by the arteries in greater quantity than is required for nutrition:—a merit of *observation, comparison, ratiocination—not of Discovery!* 4. He proved by the ligature and division of vessels that the blood passes from the heart by the arteries, and returns by the veins, but these experiments were suggested and had been partially executed by others:—a merit of *execution, confirmation—not of Discovery!* 5. *His unquestioned merits* were, however, great, viz. the exactness and soundness in his inductions; skill and diligence in his experiments; attention and delicacy in his observations; sagacity and accuracy in his reasonings; clearness and truth in his conclusions; numerous new and important reflections; steadfastness (costanza) in all his proceedings:—great merits, certainly—*but not Discovery!*"

And did Harvey really discover nothing? Dr. Zecchinelli says yes; but we appeal to his own words whether his characteristic generosity, in allowing thus much, has not got the better of his sense of justice.

"One sole DISCOVERY remained for Harvey—viz. the determining the mode of communication between the ultimate arteries and nascent veins; and even to this he himself seems not to have aspired, as he contents himself with speaking of this communication as *mediate, immediate, or in both ways.* The supposed *mediate* communication being through 'carnis porositates.' Even the term '*circulation*' is not of Harvey's *invention,* since it was used by Cæsalpinus in reference to the lesser circulation; nor is the *application* of this term to the general course of the blood *invented* by him, since it had been already made by Thomas Aquinas, when amplifying the doctrines of Aristotle."

Alas, poor Harvey!

